# The key pillars of psychosocial disability: a European perspective on challenges and solutions

**DOI:** 10.3389/fpsyt.2025.1574301

**Published:** 2025-04-07

**Authors:** Alessandra Martinelli

**Affiliations:** Unit of Epidemiological Psychiatry and Digital Mental Health, IRCCS Istituto Centro San Giovanni di Dio Fatebenefratelli, Brescia, Italy

**Keywords:** psychosocial disability, housing, employment, recovery, social inclusion, stigma, Europe

## Abstract

Psychosocial disabilities refer to a range of mental health conditions that significantly impact an individual’s ability to function in daily life and participate fully in society. Across Europe, individuals with these conditions face systemic barriers, including inadequate support services, stigma, and limited healthcare access. This perspective article examines these challenges through the lens of Maslow’s hierarchy of needs and Saraceno’s community psychiatry framework. By analyzing identified key pillars of psychosocial disability - housing, social inclusion, employment, healthcare access, service organization, and stigma – this article underscores the necessity of targeted interventions to promote dignity, autonomy, and recovery for individuals with psychosocial disabilities across Europe. Stable housing is foundational for recovery, social integration, and employment. Social inclusion and meaningful employment are essential for psychological well-being, though stigma and discrimination remain a major obstacle. Employment programs are crucial for fostering social reintegration. Healthcare access, already fragmented, can be obstacolated by stigma in healthcare settings as an additional barrier. Positive organizational culture in mental health services, emphasizing co-production and shared decision-making, is vital for recovery and healthcare access. This article highlights how key pillars of psychosocial disability are strongly interrelated, with each significantly influencing the others. The reciprocal impact among these elements demonstrates that improvements or setbacks in one area inevitably affect the others, creating either a reinforcing cycle of support or a compounding negative effect. Coordinated efforts and comprehensive strategies are essential to integrating these pillars and overcoming barriers to psychosocial disability across Europe.

## Introduction

Psychosocial disabilities refer to mental health conditions that significantly impact an individual’s ability to function in daily life, including work, education, social relationships and self-care, ultimately affecting full participation in society (1–3). Due to these challenges, individuals with psychosocial disability require comprehensive care that includes medical, social, and community support. Conditions such as schizophrenia, bipolar disorder, major depressive disorder, and other severe mental disorders can impair independent living and societal participation (4–6). Approximately 84 million people in the WHO European Region experience psychosocial disabilities (3). Across Europe, these individuals encounter numerous systemic barriers that compound their challenges, including inadequate support services, persistent stigma, and limited access to healthcare. These challenges are multifaceted and exist at multiple levels, from individual to societal (2, 7, 8).

The objective of this perspective article is to analyze the systemic barriers faced by individuals with psychosocial disabilities in Europe across six key pillars: housing, social inclusion, employment, healthcare access, organizational culture in mental health services, stigma and discrimination.

A theoretical analysis of psychosocial disabilities in Europe will be presented by applying Maslow’s hierarchy of needs (9) and Saraceno’s community psychiatry framework (10) to explore these challenges. Maslow’s model (*Maslow’s Hierarchy of Needs model*) emphasizes the necessity of meeting basic physiological and safety needs before individuals can achieve personal growth and societal contribution. Saraceno’s work in community psychiatry underscores the interconnectedness of essential life domains—family, work, and housing—in promoting mental health and social well-being. By applying Maslow’s and Saraceno’s models, the article aims to demonstrate how addressing these intertwined pillars can improve recovery, enhance social participation, and support the overall well-being of individuals with psychosocial disabilities.

## Housing

Maslow’s hierarchy of needs places shelter and security among the most fundamental requirements for human well-being (9). For individuals with psychosocial disabilities, stable housing is essential, as it supports the fulfillment of higher-order needs like social belonging and self-esteem.

Without safe housing, individuals struggle to meet basic needs, hindering personal growth and societal participation. Secure housing reduces the psychological stress associated to mental health conditions and mitigates the heightened risk of homelessness, which can exacerbate existing challenges and impede recovery. Thus, stable housing is vital for mental health and recovery (1, 7, 11, 12). Saraceno’s work further underscores the centrality of housing in recovery, emphasizing its role as a foundation for fostering social connections, supporting employment, and building pathways toward community integration (10).

Despite the importance of housing, significant disparities persist across Europe. The average number of psychiatric beds across inpatient units (psychiatric hospitals, mental health units in general hospitals, forensic facilities, community residential housing, and child/adolescent facilities) is 93 per 100,000 inhabitants, but this varies widely. In low-income countries, mental hospitals are nearly twice as large as those in high-income countries, with a median of 300 beds compared to 166. In low-income countries, the number of mental hospital beds ranges from 28 to 40 per 100,000 inhabitants. In contrast, high-income countries have the highest bed rates in psychiatric units within general hospitals (22 per 100,000 inhabitants) and in mental health community residential housing (60 per 100,000 inhabitants) (13). Notably, Italy and Iceland are the only European countries where psychiatric hospitals have been fully eliminated (14). As a result, many individuals with psychosocial disabilities remain in psychiatric hospitals rather than being integrated into their communities.

Community residential housing provides long-term or transitional living for individuals with mental health conditions, offering support, independence, and access to care from mental health professionals. In this context, Italy and England are the only countries that have developed robust mental health supported accommodation services that promote independent living. These services follow a progressive care model, where individuals gradually transition from higher to lower levels of support as they acquire skills for independent living and societal participation. This approach ensures individuals receive appropriate support tailored to their specific needs, with the goal of moving on to less supported or fully independent housing over time. While this model offers tailored support and clear goals for both staff and service users, it also requires individuals to move homes as they progress in their recovery (15, 16).

Italy, often celebrated as a pioneer of deinstitutionalization thanks to the contributions of Basaglia and the movement he inspired, exemplifies both progress and ongoing challenges (14). Basaglia’s efforts led to the closure of Italy’s psychiatric hospitals with the enactment of Law 180/1978, which dismantled institutional care in favor of community-based services (17, 18). This shift to community-based care aligns with principles championed by figures like Erving Goffman and global initiatives such as the WHO’s QualityRights Toolkit (19, 20). However, even in Italy, the deinstitutionalization process remains a work in progress (14). Recent evaluations of supported accommodation services in Italy using the Quality Indicator for Rehabilitative Care - Supported Accommodation (QuIRC-SA) tool (21, 22) revealed areas for improvement. This comprehensive tool assesses care quality across seven domains, with higher scores indicating better outcomes. The overall mean QuIRC-SA score across facilities was 52.3% (SD = 9.3) with particularly low scores in key domains such as Social Interface (48.6%, SD = 11.4) and Recovery-Based Practices (45.8%, SD = 9.1) (23). However, the QuIRC-SA domain scores for Italian supported accommodation services were lower than those of a national sample from England, except for the Treatments and Interventions domain, which was >2% higher. The mean score for England was 69.2%, with a range from 55.1% (SD = 8.4) to 86.7% (SD = 5.0), which is the only other sample for which QuIRC-SA data has been published to date (24).

Countries like The Netherlands, Finland, Sweden, and Denmark have made significant progress in balancing institutional and community-based services, and are developing alternatives such as supported accommodations and using the “Housing First” approach, enabling individuals with psychosocial disabilities to live independently and participate fully in society (14, 25, 26).

These findings underscore the need for sustained investment in rehabilitative housing programs that prioritize not only physical shelter but also social reintegration and recovery-focused practices. Political commitment is essential to reduce institutionalization and promote a human rights-based approach to housing for individuals with psychosocial disabilities.

## Social inclusion

Maslow’s model highlights the importance of belonging and social connection for psychological well-being (9). Saraceno further emphasized that supportive social networks mitigate isolation and promote resilience (10). Social inclusion fosters a sense of identity, purpose, and connection, which are essential for recovery (8, 27). Despite these insights, individuals with psychosocial disabilities often face stigma and discrimination, leading to social isolation and diminished self-worth (28).

Data from Eurostat indicate that in 2022, people with disabilities in the EU had lower participation rates in cultural activities, sporting events, and voluntary work compared to those without disabilities. For example, only 10.3% of people with disabilities participated in formal voluntary activities, compared to 13.0% of those without disabilities. The highlighted disabilities gap varies significantly by country. In 2022, the percentage of people (16 and older) who visited the cinema, attended a live performance, or explored a cultural site in the past year was the highest in Luxembourg (77.6%) and Denmark (77.1%) and the lowest in Romania (22.2%) and Bulgaria (19.7%). Romania had the biggest gap, where 28.3% of people without disabilities took part in cultural activities, compared to just 7.1% of those with disabilities. In 2022, the percentage of people (16+) meeting with family at least once a year ranged from 93.6% in Estonia to 99.4% in Poland and Romania. In most EU countries, people with disabilities were less likely to do so, except in Cyprus, where their rate was 1.8 percentage points (pp) higher. The largest disability gap was in Estonia (6.6 pp). Women were generally more likely than men to meet family. For meeting friends at least once a year, the rates varied more, from 78.5% in Latvia to over 98% in Denmark, Cyprus, Greece, Croatia, and Bulgaria. Again, people with disabilities participated less, with the biggest gaps in Malta (20.9 pp), Estonia (20.1 pp), and Latvia (18.2 pp). Unlike family gatherings, men were more likely than women to meet friends. Participation in voluntary activities also showed a disability gap. In 2022, 12.3% of EU citizens took part in formal volunteering (10.3% for people with disabilities, 13% for those without). Informal volunteering had a smaller gap (13.3% vs. 14.7%). Active citizenship (e.g., political activities) was reported by 7.4% of people with disabilities and 8.4% of those without. Gender gaps in volunteering and civic activities were minor, but disability gaps varied by age. Younger people with disabilities had higher participation rates than their peers without disabilities, while older individuals (65+) with disabilities participated significantly less than their non-disabled counterparts (29). This variability suggests that while there are common challenges, specific conditions and policies differ widely across Europe (30). However, there is a lack of specific, detailed data on psychosocial disabilities across Europe. This is partly due to the absence of a unified definition of disability and inconsistent data collection methods across countries.

Across Europe, various initiatives have been introduced to boost community involvement for individuals with psychosocial disabilities and support their independence. These include cultural, educational, and recreational programs, as well as peer support networks (31), that have proven effective in reducing stigma and fostering inclusive environments (32, 33). Family education programs (34, 35), such as those implemented in the United Kingdom, help build empathy and understanding, creating nurturing environments that support recovery (31, 36–40).

## Employment

Meaningful work fulfills higher-level needs in Maslow’s hierarchy, contributing to self-esteem and self-actualization (9). Saraceno’s work emphasized the reciprocal relationship between employment and mental health, noting that meaningful work reduces stress and enhances social participation (10). However, workplace discrimination and misconceptions about the capabilities of individuals with psychosocial disabilities continue to limit employment opportunities (41, 42).

Individuals with disabilities, including those with psychosocial disabilities, face higher risks of poverty and social exclusion. In the EU, nearly 30% of people with disabilities live in poverty, which is significantly higher than those without disabilities (43). The economic burden of mental health disorders in the EU is substantial, accounting for up to 4% of GDP annually, or over €600 billion (44). In Europe, only 10% to 20% of people with severe mental disorder are employed, and they are twice as likely to lose their jobs after the onset of their condition (45–47). From 2014 to 2022, the employment gap between individuals with and without disabilities in the EU27 averaged between 22.7 and 21.4 percentage points. This gap varies significantly across EU countries, with Ireland having one of the largest at nearly 40 percentage points, while Luxembourg has one of the smallest at 8.5 percentage points. Belgium, Bulgaria, and Croatia also experience substantial disparities (48). People with psychosocial disability living in community residential housing or supported accommodation face even greater challenges in finding employment. For example, in Italy, 75.5% of residents in supported accommodation services are unemployed (23, 49).

Supported employment programs, social enterprises, and models like Individual Placement, Support (IPS) and Clubhouse have demonstrated success in improving employment outcomes and supporting people with psychosocial disability in their recovery (50–52). In the WHO European Region, 91% of countries have reported having at least one stand-alone mental health policy or plan related to social protection, employment, education, or other areas (40).

The economic and social benefits of inclusive employment cannot be overstated—reducing economic insecurity, fostering social connections, and promoting mental well-being (39, 53, 54).

## Healthcare access

Access to healthcare is a critical pillar for individuals with psychosocial disabilities. Maslow’s model underscores the importance of health and safety (9), while Saraceno’s work highlights the need for integrated and community-based mental health services (10). Despite efforts to improve healthcare access, barriers persist across Europe (40).

Although mental health services and psychotropic medicines in the WHO European Region are fully covered in 100% and 98% of cases or require at most a 20% co-payment (40), many individuals still struggle to receive care. Stigma, discrimination in healthcare settings, and social factors—such as precarious employment and broader societal biases—often discourage individuals from seeking help. These challenges not only affect mental health but also hinder access to integrated care, worsening inequalities and health outcomes (31).

To address these issues, Global Target 2.3 of the Comprehensive Mental Health Action Plan (54) aims for 80% of countries to integrate mental health into primary healthcare by 2030. This initiative promotes a shift from long-stay mental hospitals to community-based care. In the WHO European Region, 74% of countries have reported having guidelines for this integration, with pharmacological interventions in 71% and psychosocial interventions in 30%. Additionally, government social support for individuals with mental health conditions varies widely across WHO European countries, ranging from 2% to 50%. Despite this effort, health systems across Europe still struggle to meet the demand for mental health care. Between 35% to 50% of individuals with severe mental disorders in high-income countries go untreated, while the gap is likely even wider in lower-income regions (40). Stigma within healthcare settings and fragmented care systems further prevent individuals from receiving comprehensive treatment (31).

Ensuring that mental health care is affordable, accessible, and culturally sensitive is essential for promoting recovery and reducing health disparities (54–56).

## Organizational culture

Saraceno’s emphasis on the organizational culture of mental health services underscores its critical role in shaping recovery outcomes (10). Positive organizational environments foster collaboration, respect, and shared decision-making between professionals, patients, and families. In contrast, hierarchical and rigid systems often hinder engagement and recovery. A shift toward recovery-oriented practices that prioritize personal goals and autonomy is essential (57–59). Co-production models, where individuals with lived experience collaborate in service design and delivery, have shown promise in fostering more inclusive and effective care environments (4, 60). Identifying European countries adopting recovery-oriented practices is complex due to overlaps with broader mental health strategies. While many demonstrate commitment, implementation varies based on contextual and resource factors. Sustained efforts are crucial for accessible, recovery-focused care (42, 61–63).

## Stigma

Despite Maslow and Saraceno not directly addressing stigma and discrimination, their models cannot be fully realized without tackling these barriers. Stigma and discrimination must be addressed to fully realize the potential of Maslow and Saraceno’s models. These barriers hinder access to services, reduce employment opportunities, and contribute to social exclusion (28, 64, 65). Despite international frameworks like the Convention on the Rights of Persons with Disabilities (CRPD) advocating for the elimination of stigma (8), progress remains insufficient (31).

Stigma and discrimination surrounding mental health persist in many European countries, hindering individuals from seeking help (44). Around a quarter (24.7%) of respondents across the EU-27 reported difficulty speaking to a person with a significant mental disorder, indicating a significant social distance (66).

Initiatives like the International Study of Discrimination and Stigma Outcomes (INDIGO) Network projects emphasize the need for public awareness, anti-discrimination laws, and better training for mental health professionals (67).

## Discussion

This article seeks to analyze the systemic barriers faced by individuals with psychosocial disabilities in Europe, focusing on six key pillars: housing, social inclusion, employment, healthcare access, organizational culture, and stigma. By applying Maslow’s and Saraceno’s models, the findings underscore how these key pillars are strongly interrelated, with each significantly influencing the others, reinforcing the necessity of integrated interventions. The reciprocal impact among these elements demonstrates that improvements or setbacks in one area inevitably affect the others, either fostering a reinforcing cycle of support or exacerbating existing challenges. For example, stable housing provides a foundation for employment and social inclusion, while access to meaningful work enhances self-esteem and promotes financial independence, reducing economic and psychological stress. Similarly, healthcare access is vital for managing mental health conditions, yet stigma and fragmented services often prevent individuals from seeking the care they need, ultimately affecting their ability to participate in society. **Table 1** illustrates how integrating Maslow’s and Saraceno’s models into policy and practice can create a comprehensive framework for addressing these barriers in a structured approach and fostering long-term recovery. Maslow’s model emphasizes the progression from basic needs to self-actualization, reinforcing the importance of housing, employment, and healthcare as foundational to recovery. Saraceno’s contributions further stress the significance of social inclusion, community-based care, and supportive environments in fostering autonomy and dignity.

**Table 1:** Integration of Maslow’s hierarchy of needs and Saraceno’s recovery model to address psychosocial disability.

Pillar	Maslow’s Contribution	Saraceno’s Contribution	Impact on Psychosocial Disability
**Housing**	Shelter and security are fundamental for well-being.	Housing is central to recovery, fostering social connections, employment, and integration.	Secure housing reduces stress, prevents homelessness, and promotes recovery. Political commitment is needed to ensure human rights-based housing.
**Social Inclusion**	Belonging and social connection are essential for psychological well-being.	Supportive social networks reduce isolation and enhance resilience.	Reducing stigma and discrimination fosters identity, purpose, and connection, improving recovery outcomes.
**Employment**	Meaningful work fulfills self-esteem and self-actualization needs.	Employment and mental health have a reciprocal relationship—work enhances social participation and reduces stress.	Inclusive employment reduces economic insecurity, strengthens social ties, and supports mental well-being.
**Healthcare Access**	Health and safety are foundational to well-being.	Integrated, community-based care is essential for effective mental health services.	Accessible, affordable, and culturally sensitive healthcare promotes recovery and reduces health disparities.
**Organizational Culture**	–	Organizational culture in mental health services shapes recovery outcomes.	Transforming rigid, hierarchical systems into inclusive, patient-centered models enhances engagement and well-being.
**Stigma**	–	–	Tackling stigma is crucial for access to services, employment opportunities, and social inclusion.

Furthermore, the interconnected pillars shape recovery, social participation, and overall well-being and their development requires integrated policies and interventions. Governments, healthcare systems, and communities must collaborate to invest in rehabilitative housing, inclusive employment, and community-based mental health services. Reducing stigma requires systemic reforms and public awareness efforts to foster inclusion.

Future research should assess the long-term impact of policies based on Maslow’s and Saraceno’s models, evaluating their effectiveness in employment, social participation, and mental health outcomes. Comparative studies across European regions can help identify adaptable best practices.

By refining and applying the models of Maslow and Saraceno, it becomes evident that addressing the pillars of psychosocial disability is essential for fostering recovery, independence, and dignity. Housing, social inclusion, employment, healthcare access, organizational culture, and stigma are interconnected domains that require sustained investment and collaboration among governments, healthcare systems, and communities. Only through such a holistic approach can we create a society where individuals with psychosocial disabilities can thrive, free from discrimination and exclusion.

## Data Availability

The original contributions presented in the study are included in the article/supplementary material. Further inquiries can be directed to the corresponding author.
